# Factors Affecting Mealtime Difficulties in Older Adults with Dementia Living in Long-Term Care Facilities: A Multilevel Model Analysis

**DOI:** 10.1155/2023/4427390

**Published:** 2023-03-03

**Authors:** Dukyoo Jung, Jennie C. De Gagne, Hyesoon Lee, Leeho Yoo, Jisung Park, Eunju Choi

**Affiliations:** ^1^College of Nursing, Ewha Womans University, Ewhayeodae-gil, Seodaemun-gu, Seoul 03760, Republic of Korea; ^2^School of Nursing, Duke University, 307 Trent Drive, DUMC 3322, Pearson Building, Durham 27710, USA; ^3^Department of Nursing, Semyung University, Jecheon-si, Chungbuk, Republic of Korea; ^4^College of Nursing, Ewha Womans University, 52, Ewhayeodae-gil, Seodaemun-gu, Seoul 03760, Republic of Korea

## Abstract

**Background:**

Mealtime difficulty in this population should be examined from an extensive perspective, rather than approaching it as an individual problem. *Method(s)*. This was a cross-sectional study involving 342 participant dyads from 57 long-term care facilities; 114 direct care workers; and 342 older adults with dementia. A multilevel regression analysis was conducted using the MPlus 8.0 program.

**Results:**

Among intrapersonal factors, age, cognitive function, activities of daily living, and number of beds in the facility were identified as affecting mealtime difficulty. Environmental factors included meal assistant education experience and whether an environment suitable for eating had been established. *Conclusion(s)*. The results show that intrapersonal factors exert a large influence on the mealtime difficulties of older adults with dementia in long-term care facilities and support the need to improve environmental factors, which are modifiable. *Implications for Nursing Management*. This study provided useful information about the influence of leaders on mealtime difficulties in older adults with dementia. Leaders should establish an environment in the facility for reduced mealtime difficulties.

## 1. Introduction

Mealtime difficulties in older adults with dementia are defined as frustrations or problems that arise during mealtimes [1, 2] and generally occur in individuals in the middle or late stages of dementia [3]. They gradually experience difficulty picking up food with utensils, putting it in their mouth, chewing, and swallowing. Eventually, in the late stages of dementia, swallowing quality appears to fail [4]. Older adults with dementia and mealtime difficulties experience symptoms such as reduced nutritional intake, weight loss, dehydration, and aspiration [5]; secondary weight loss increases the incidence of myasthenia, bedsores, and immobility [4].

The social-ecological model helps to determine the complex interplay of factors affecting mealtime difficulties experienced by older adults with dementia [6, 7] in which it can be used to identify influences on eating performance from a multilevel perspective, encompassing their intrapersonal-, interpersonal-, environmental-, and policy-related aspects [8–10]. Intrapersonal factors affecting mealtime difficulty include age, cognitive function, physical ability, complications, behavioral and psychological symptoms of dementia, and the individual perspectives of older adults with dementia [11–14]. Interpersonal factors consist of the knowledge, attitudes, and behaviors contributing to direct care workers' interactions [7, 12, 15]. Environmental factors include aspects of the physical environment (e.g., type of meal, how food is served, and appropriate staffing levels) that might affect mealtime difficulties [11, 13, 16, 17] as well as care facility factors such as work-oriented policies and provision of appropriate accommodations [12, 18].

As such, the factors that influence mealtime difficulties in older adults with dementia can be multifaceted. Therefore, based on the social-ecological model, it is necessary to address older adults with dementia, caregivers, and environment levels to identify factors that may be associated with mealtime difficulties. Multilevel models allow for the examination of how individual characteristics, such as cognitive impairment and functional ability, interact with group level factors, such as staffing levels, and the care environment to predict the outcome of mealtime difficulties [19]. This type of analysis provides a more comprehensive understanding of the complex and dynamic factors that contribute to mealtime difficulties in older adults with dementia in long-term care facilities.

Multilevel model analysis is used to study mealtime difficulties in older adults with dementia living in long-term care facilities because it takes into account both individual and group level factors that may influence the outcome of interest [13, 20]. In previous studies [13, 20], the effects of individual and environmental characteristics on mealtime performance were confirmed by multilevel analysis, and the direct care worker factor was analyzed at the environmental level. However, care workers play an essential role in providing eating assistance for older adults with dementia, who depend highly on help from others to accomplish their activities of daily living [21]. The direct caregiver factor is important because it is a factor that can be more easily modified and adjusted than other factors to alleviate mealtime difficulties [12, 21]; therefore, it is necessary to analyze interpersonal factors independently when assessing the mealtime difficulties of older adults with dementia. In addition, the reflection of cultural factors in mealtime difficulties, including the influence of sociocultural contexts and care facility policies, is crucial; however, there is a lack of research that addresses this issue specifically in the Korean context.

## 2. Methods

### 2.1. Design

This study used a multilevel cross-sectional design to determine the relationship between intrapersonal-, interpersonal-, and environmental-level factors and the mealtime difficulties of older adults with dementia in long-term care facilities and to identify which aspects of the factors are influential.

### 2.2. Participants

Following Maas and Hox [22] criteria, as the group level sample size is statistically more important than the individual-level sample size, Schoeneberger [23] proposed recruiting over 30 groups of 10 or more individual subjects.

The long-term care facilities participating in this study were categorized based on the total number of beds (less than 30, more than 30 but less than 50, 50 to 100, and more than 100) and the results of regular evaluation scores (A, B, C, D, or E) by the Korean National Insurance Corporation. Sixty institutions that agreed to participate in the study were selected based on a dropout rate of 20%.

According to the literature review, the number of analysis levels was three. The number of participating long-term care facilities was 60 (Level 3); the number of participating direct care workers (care workers who provide hands-on assistance, support, and care to older adults with dementia in long-term care facilities) was 120 (Level 2), twice the number of long-term care facilities; and the number of older adults with dementia who participated was 360 (Level 1), three times the number of direct care workers. Direct care workers were matched with two older adults under their care so we could observe and record any mealtime difficulties encountered.

A preliminary survey was conducted to measure response time and check whether any questions were not understood after the survey. The participants in this preliminary survey comprised two long-term care facilities (Level 3), four direct care workers (Level 2), and 12 older adults with dementia (Level 3). The current study participants comprised 342 dyads from 57 long-term care facilities, 114 direct care workers, and 342 older adults with dementia. Data excluding attrition of 5% (insufficient response) were used for the final analysis.

### 2.3. Variables and Measures

#### 2.3.1. Level 1: Intrapersonal Factor

A 37-item tool related to cognitive impairment currently used in nursing homes was used to measure elements such as reduced orientation, judgment, attention, and concentration, with a higher score indicating lower cognitive function. The functional independence measure was used to measure seven items related to eating-related activities of daily living, with a higher score indicating more independence. The mealtime difficulty scale for older adults with dementia was used to measure mealtime difficulty [24], with higher scores indicating greater mealtime difficulty. In this study, we modified any negative words used that might have confused survey participants. At the time of development, Cronbach's *α* was 0.91; in this study, Cronbach's *α* was 0.92.

#### 2.3.2. Level 2: Interpersonal Factor

The survey of direct care workers included general characteristics of age, gender, level of education, work experience, duty, attitude, and knowledge about feeding. Attitudes toward feeding were measured using the Formal Direct Care Workers' Attitude toward Feeding Dementia Patients Questionnaire, developed by Chang and Roberts [11] and translated by Hong and Gu [25]; the higher the score, the more negative the attitude towards feeding. In Hong and Gu [25], Cronbach's *α* was 0.73; in this study, Cronbach's *α* was 0.83. In addition, knowledge about feeding was assessed using the Formal Direct Care Workers' Knowledge of Feeding Dementia Patients Questionnaire, developed by Chang and Roberts [11] and translated by Hong and Gu [25], which has a total of 21 questions. The higher the value, the higher the level of knowledge. In a study by Hong and Gu [25], Kuder-Richardson (KR) was 20 = 0.63; in this study, KR-20 = 0.63.

#### 2.3.3. Level 3: Environmental Factor

Organizational-level variables were investigated for the head of the nursing facility, and characteristics related to mealtime difficulty at the facility (year of establishment, facility operator, certificate possessed by the facility manager, number of nurses in the facility, number of nursing assistants in the facility, number of direct care workers in the facility, facility residents, whether regular education was performed, facility size, need for food support education, and environmental factors related to difficulty in eating) were investigated.

### 2.4. Statistical Analysis

The collected data were verified for statistical significance based on *p* < 0.05 using SPSS 25.0, Microsoft Office Excel, and MPlus 8.0 [26]. Descriptive statistical analysis was used to describe each variable, and Cronbach's *α* was used to assess the reliability of the research measurements. A three-level random intercept model was used to analyze the factors influencing mealtime difficulties experienced by older adults with dementia in long-term care facilities. The multilayer model estimation method of Mplus was used for estimation, and the full-information maximum likelihood (FIML) method, which is known to be robust even in violation of the statistical assumptions of the data, was used as well. The *z*-test, which is the Mplus estimation method, was performed for the parameter test.

### 2.5. Ethical Considerations

This study was conducted after OOOO University's Institutional Review Board approved the research ethics (OOOO-202203-0007-01). All participants were provided with sufficient information about the study and instructed that they could withdraw from the study at any time. All data were kept confidential.

## 3. Results

### 3.1. Participants' Characteristics

Table 1 shows the general characteristics of the participants in long-term care facilities. There was 342 Level 1 participants (older adults with dementia), with an average cognitive function of 15.25 ± 7.12 and activities of daily living of 4.64 (all within the middle range). The average mealtime difficulty in older adults with dementia was 34.49 ± 11.34. There were 114 Level 2 participants (direct care workers). The average attitude toward feeding was 7.46 ± 10.85, and the average knowledge of feeding was 11.14 ± 3.26. There were 57 Level 3 participants (the heads of the long-term care facilities). Twenty-two facilities had 25–50 beds (28.6%), accounting for the largest proportion. Forty-seven (82.5%) of the facilities had meal assistant education experience, and most facilities (*n* = 56, 98.3%) required meal assistant education experience.

### 3.2. Multilevel Analysis

Level 1 (intrapersonal factors) and Level 3 (environmental factors) were statistically significant for mealtime difficulties in older adults with dementia, with the explanatory powers being 53.7% for Level 1 and 23.0% for Level 3. The explanatory power of the model incorporating Levels 1, 2, and 3 was 39.7%. Level 1 most significantly explained mealtime difficulties. As a result of the multilevel analysis, age, cognitive function, activities of daily living, the total number of beds in the facility, meal assistant education experience, and the number of suitable environments for meals were identified as factors affecting mealtime difficulties in older adults with dementia. The analysis results for each model are as follows.

#### 3.2.1. Model 1

Table 2 presents the results of the three-level multilevel regression model. There was a difference between intrapersonal and environmental factors in the mealtime difficulty score, which is the dependent variable. As the result of estimating the intraclass correlation coefficient (ICC), the Level 2 ICC estimate was found to be 0.008, which can be explained by the fact that only 0.8% had mealtime difficulties due to the interpersonal factor difference. The Level 3 ICC estimate was found to be 0.489, which means that 48.6% of mealtime difficulties occurred because of the difference in environmental factors. The degree of mealtime difficulty experienced differs depending on the environmental factors, a characteristic of the group; therefore, the necessity of multilayer analysis was confirmed.

#### 3.2.2. Model 2

Model 2 predicted that older adults with dementia, lower cognitive function, and activities of daily living would experience more mealtime difficulty. Moreover, it was predicted that males would experience more mealtime difficulty. The residual variance at Level 1 was 53.65, which was lower than that at 65.12, which means that the selected Level 1 predictors sufficiently explain the variability in the dependent variable (mealtime difficulty).

#### 3.2.3. Model 3

Among the Level 2 independent variables, gender, cognitive function, and activities of daily living were statistically predicted to significantly impact mealtime difficulties. While controlling for Level 1 predictors, no statistically significant predictors of mealtime difficulties were added among the Level 2 predictors. This may be because the Level 2 variance (*r*^2^) was very small (0.987) and not statistically significant (*p* = 0.871), so there was little difference in mealtime difficulties due to variance in direct care workers.

#### 3.2.4. Model 4

Level 1 *R*^2^ was 0.309, and the predictors explained 30.9% of the variability in the Level 1 dependent variable (mealtime difficulty). Among the Level 2 predictors, no statistically significant predictors of mealtime difficulties existed. At Level 2, *R*^2^ was 0.368, and the predictors explained 36.8% of the variability in the Level 2 dependent variable (mealtime difficulty). In addition, this model confirmed the statistical significance of Level 3, Level 1, and Level 2 predictors in the final model of the study. It was predicted that a group with meal assistant education, a large facility size, and a few suitable environments for meals would have a higher likelihood of experiencing mealtime difficulty at Level 3 while controlling Level 1 and Level 2 predictors. By controlling for Level 1 and Level 2 predictors, at Level 3, the group with meal assistant education experienced more mealtime difficulties, and the larger the facility size, the smaller the number of suitable environments for meals and the more mealtime difficulty encountered. The predictors accounted for 30.9% of the variability in the Level 3 dependent variable (mealtime difficulty), with *R*^2^ = 0.309 at Level 3. In addition, the multicorrelation square of the entire model integrating all levels was calculated [19], and the multicorrelation square *R*^2^ of Model 4 was 0.397. This is larger than Model 1 (0%), Model 2 (29.1%), Model 3 (33.4%), and Model 4 (39.7%), which explains its largest variability in mealtime difficulties.

## 4. Discussion

This study aimed to conduct a multilevel analysis of mealtime difficulties in older adults with dementia based on the social-ecological model. According to this model, mealtime difficulty variables were classified into and analyzed as three levels: Level 1 (intrapersonal), Level 2 (interpersonal), and Level 3 (environment). Among these, the level that most affected the degree of mealtime difficulties experienced was Level 1. At Level 1, sex, cognitive function, and activities of daily living influenced mealtime difficulties. At Level 3, the degree of meal assistant education, the size of the facility, and the number of suitable environments for meals at the environmental level influenced mealtime difficulties. However, no variable predicted mealtime difficulties at Level 2.

The findings of this study suggest that cognitive function and activities of daily living play a significant role in mealtime difficulties. Individuals with lower cognitive function may find it more challenging to eat them, whereas those with higher abilities in performing activities of daily living may experience less challenges. The results are similar to those of previous studies [7, 27]. This may be because eating dependence increases as cognitive function and physical function decreases [28]. In addition, the study indicates that women may experience more mealtime difficulties than men and it is similar with some previous studies [29, 30]. This is because these results may occur due to changes in dental conditions according to gender [27]. However, since there was no difference in gender in most of the studies [7, 31], further studies are needed on the effect of additional intrapersonal factors, including dental characteristics, depression, and polymedications on mealtime difficulties [32, 33].

The lower the cognitive function is, the more difficult it is to eat independently, as shown in previous research [7, 28]. Because the older adults with dementia have difficulty describing their intentions, care workers should be trained to recognize and manage various expressions of mealtime difficulties. In a previous study on mealtime difficulties in older people, the lower the daily living activity is, the more difficult it was to eat. The same results were also found in this study [7, 28, 31]. This may be due to the deterioration of physical function as dementia progresses, thus limiting the range of movement needed to use eating utensils [11, 34]. A pre-emptive approach is needed to prevent the deterioration of physical function, including the eating function of older people with dementia. Direct care workers need to encourage and help residents to eat meals independently rather than providing unconditional mealtime assistance.

The mealtime difficulties of older adults with dementia were greater in the group in which direct care workers received mealtime difficulty education. This result differs from the general expectation that mealtime difficulty education would result in fewer difficulties experienced during mealtimes. In this study, however, it is notable that although the participating facility provided training, 47.4% of the direct care workers in the facility scored less than 50% in tests to demonstrate their feeding-related knowledge. Even though feeding training was conducted, the knowledge of direct care workers did not improve, and mealtime difficulties were subsequently not reduced. This result may be that direct care workers tend to rely on habit or experience rather than education [35]. Nevertheless, previous studies have proven that direct care workers' knowledge and attitudes affect mealtime difficulties experienced by those they assist [2, 11]. Thus, continuous education that improves the quality of care provision is necessary to reduce mealtime difficulties.

The current results showed that the larger the size of the facility is, the more mealtime difficulties were experienced. This is related to previous studies that the proportion of residents with high nursing demands is high in large-scale facilities [36]. The high nursing demand means that the ability to perform daily life is low [37], and the lower the ability to perform daily life, the more difficult it is to eat [12]. Therefore, mealtime difficulties might increase in large-size facilities with many people with low daily performance. In the previous study, the higher the nursing demand is, the more direct care workers are needed [38]. Therefore, proper staffing is necessary to adequately assist older adults with high nursing demands and difficulties in mealtime due to low ADL. Likewise, in a recent qualitative study of direct care workers, inadequate staffing was found to be a barrier to mealtime assistance [20].

Preparing an appropriate mealtime environment is essential to prevent or address eating difficulties. The appropriate environment presented in this study included providing adequate food and utensils regarding individual preference or eating abilities, proper space for dining (noise, light, odor, and furniture), and enough time to eat, and mealtime was taken together with other residents. Slaughter et al. [14] proposed that residents can eat without difficulty by using “finger food,” no-spill cups, appropriate utensils, and plate guards as assistive eating devices and customizing mealtimes based on food preferences and swallowing function. A brighter and quieter dining environment might prevent residents with dementia from being distracted by environmental factors and alleviate their sundowning syndrome [11, 31]. In Korea, long-term care facilities are evaluated every 3 years. The evaluation only considers items related to “pleasant environments” and “falls prevention.” Guidelines for the care environment that consider the needs of older adults with dementia, including those related to dining and meals, have not been presented. Therefore, developing guidelines to maintain an appropriate environment to reduce mealtime difficulties for residents with dementia is necessary.

Lastly, at Level 2, no variable was found that predicted mealtime difficulties. Direct care workers' knowledge and attitudes were reported as influential factors in mealtime difficulties [7, 12, 15]. In this study, however, only 0.8% of the total change was explained at the interpersonal level, perhaps because the knowledge and attributes of the direct care workers who participated were distributed with low variance in the low score range, and there were no significant results in the multilevel analysis; therefore, to confirm the effect of Level 2 factors on meal difficulties, direct care workers must be analyzed again after improving their knowledge and attitudes to some extent through continuing qualitative meal difficulty education.

## 5. Limitations

This study has several limitations. First, the cross-sectional nature of the data limits the interpretation of causality. Second, variables related to interpersonal and environmental factors are sensitive to sociocultural influences, so caution is required when generalizing to other countries. Third, the institutional-grade was not used as an environmental variable because a participating institution had not yet been officially graded at the time of the study due to severe acute respiratory syndrome coronavirus 2 restrictions. Hence, a study that considers the institutions' quality grading is necessary. Finally, studies on other possible influencing factors are needed because we considered only a limited number of factors related to eating difficulties. In particular, interpersonal factors require consideration based on actual observations. A strength of this study is that quota sampling was performed that considered region, institution grade, and the number of beds. Theoretically, we identified factors related to eating difficulties in older people with dementia using robust sample size and at various levels.

## 6. Conclusions

Our study demonstrates the effects of intrapersonal, interpersonal, and environmental factors on eating difficulties in older people with dementia. Our findings confirm that intrapersonal factors significantly influence the eating difficulties experienced by older adults with dementia in long-term care facilities and support the need for an approach that considers the environmental factor, which is modifiable [39–42].

## Figures and Tables

**Table 1:** Participant characteristics.

Characteristics	Categories	Total *n* (%) or M ± SD	Range
*Level 1 (older adults with dementia* *=* *342)* *Intrapersonal factor*			
Age		84.08 ± 7.12	60–104
Gender	Male	79 (23.1)	
	Female	263 (76.9)	
Cognitive function		15.25 ± 7.12	0–32
Activities of daily living		4.64 ± 1.82	1–7
Period of institutionalization		3.22 ± 2.58	0–21
Mealtime difficulty		34.49 ± 11.34	19–78

*Level 2 (direct care workers* *=* *114)* *Interpersonal factor*			
Level of education	<Middle school	13 (11.41)	
	High school	78 (68.42)	
	≥University	23 (20.18)	
Duty	Fixed	20 (17.54)	
	8-hour shift	26 (22.81)	
	12-hour shift	65 (57.02)	
	24-hour	3 (2.63)	
Current work experience (years)		4.52 ± 4.27	
Attitude toward feeding		73.46 ± 10.85	43–100
Knowledge toward feeding		11.14 ± 3.26	4–19

*Level 3 (long-term care facility* *=* *57)* *Environmental factor*			
Length of the facility establishment		12.09 ± 4.63	4–26
Facility type	Private	24 (42.1)	
	Corporate	33 (57.9)	
Facility size		55.88 ± 44.23	13–258
	≤25 beds	4 (7.0)	
	25–50 beds	22 (38.6)	
	50–75 beds	11 (19.3)	
	75–100 beds	14 (24.6)	
	≥100–200 beds	6 (10.5)	
Number of suitable environment for meals		4.68 ± 2.69	1–10
Meal assistant education experience	Yes	47 (82.5)	
	No	10 (17.5)	
Meal assistant education requirements	Very required	23 (40.4)	
	Required	33 (57.9)	
	Not required	1 (1.8)	
Number of nurses		0.56 ± 1.35	0–8
Number of nurse assistants		2.46 ± 1.81	0–10
Number of direct care workers (≥6 month)		15.96 ± 12.03	2–57

**Table 2:** Multilevel analysis of the four fitted models on mealtime difficulties in older adults with dementia.

Parameters		Classifications		*Model 1*		*Model 2*		*Model 3*		*Model 4*	
				Estimate	*ρ*	Estimate	*ρ*	Estimate	*ρ*	Estimate	*ρ*
Fixed effect		Intercept		34.49	<0.001	34.49	<0.001	34.49	<0.001	34.48	<0.001
		Age	*Level 1*			−0.04	0.492	−0.04	0.029	−0.02	0.037
		Gender (ref = men)				2.23	0.024	2.13	0.563	2.08	0.705
		Cognitive function				0.44	<0.001	0.40	<0.001	0.38	<0.001
		Activities of daily living				−1.93	<0.001	−1.94	<0.001	−1.91	<0.001
		Period of institutionalization				0.21	0.178	0.20	0.192	0.21	0.178
		Current work experience (years)	*Level 2*					−0.08	0.569	−0.05	0.741
		Attitude toward feeding						−0.07	0.276	−0.04	0.548
		Knowledge toward feeding						−0.36	0.124	−0.22	0.360
		Duration of the facility establishment	*Level 3*							−0.26	0.183
		Facility type								−0.10	0.958
		The total number of beds in facility								1.91	0.009
		Nurse ratio to admission capacity								−1.11	0.089
		Care worker ratio to admission capacity								0.02	0.730
		Meal assistant education experience (ref = no)								5.10	0.005
		Number of suitable environments for meals								−0.74	0.010

*Random effect*											
Variance at level 1, *σ*^2^				65.12	<0.001	53.65	<0.001	53.82	<0.001	52.73	<0.001
Variance at level 2 *τ*^2^				0.99	0.871	0.03	0.994	0.29	0.925	1.83	0.613
Variance at level 3, *ψ*^2^				34.49	<0.001	37.48	<0.001	31.48	<0.001	22.98	0.002
*R*1^2^						0.334		0.316		0.309	
*R *_2_^2^								0.919		0.368	
*R *_3_^2^										0.309	
*R * ^2^				0		0.291		0.334		0.397	

## Data Availability

The [research] data used to support the findings of this study are restricted by the [Ewha Research Ethics and Compliance Online System] in order to protect [PATIENT PRIVACY]. Data are available from [Eunju Choi, celestial_@naver.com] for researchers who meet the criteria for access to confidential data.

## Additional Points

Nurse managers should consider improving the quality of education and providing staff education to reduce mealtime difficulty in older adults with dementia. Furthermore, it is essential that different levels of adequate nursing staff be set according to facility size. Nurse managers must identify individual needs and preferences to create and implement an environment that can reduce mealtime difficulties. In addition, nursing managers should position more direct care workers so that residents can assist with meals adequately.
